# Functional autoantibodies against G protein-coupled receptors in hepatic and pulmonary hypertensions in human schistosomiasis

**DOI:** 10.3389/fimmu.2024.1404384

**Published:** 2024-06-17

**Authors:** Fernando Antonio Botoni, José Roberto Lambertucci, Robson Augusto Souza Santos, Johannes Müller, Andre Talvani, Gerd Wallukat

**Affiliations:** ^1^ Postgraduate Program in Infectiology and Tropical Medicine, School of Medicine, Universidade Federal de Minas Gerais, Belo Horizonte, Brazil; ^2^ Internal Medicine Department, School of Medicine Universidade Federal de Minas Gerais, Belo Horizonte, Brazil; ^3^ Fundação Hospitalar do Estado de Minas Gerais - FHEMIG, Belo Horizonte, Brazil; ^4^ Postgraduate Program in Health and Nutrition, School of Nutrition, Universidade Federal de Ouro Preto, Ouro Preto, Brazil; ^5^ Department of Physiology and Biophysics of the Institute of Biological Sciences, Universidade Federal de Minas Gerais, Belo Horizonte, Brazil; ^6^ Berlin Cures GmbH, Berlin, Germany; ^7^ Department of Biological Sciences, Universidade Federal Ouro Preto, Ouro Preto, Brazil; ^8^ Max-Delbrück Centrum für Molekulare Medizin, Berlin, Germany

**Keywords:** *Schistosoma mansoni*, GPCR, inflammation, α1adrenoceptor, pulmonary hypertension, endothelin-1, angiotensin II

## Abstract

**Introduction:**

Schistosomiasis (SM) is a parasitic disease caused by *Schistosoma mansoni*. SM causes chronic inflammation induced by parasitic eggs, with collagen/fibrosis deposition in the granuloma process in the liver, spleen, central nervous system, kidneys, and lungs. Pulmonary arterial hypertension (PAH) is a clinical manifestation characterized by high pressure in the pulmonary circulation and right ventricular overload. This study investigated the production of functional autoantibodies (fAABs) against the second loop of the G-protein-coupled receptor (GPCR) in the presence of hepatic and PAH forms of human SM.

**Methods:**

Uninfected and infected individuals presenting acute and chronic manifestations (e.g., hepatointestinal, hepato-splenic without PAH, and hepato-splenic with PAH) of SM were clinically evaluated and their blood was collected to identify fAABs/GPCRs capable of recognizing endothelin 1, angiotensin II, and a-1 adrenergic receptor. Human serum was analyzed in rat cardiomyocytes cultured in the presence of the receptor antagonists urapidil, losartan, and BQ123.

**Results:**

The fAABs/GPCRs from chronic hepatic and PAH SM individuals, but not from acute SM individuals, recognized the three receptors. In the presence of the antagonists, there was a reduction in beating rate changes in cultured cardiomyocytes. In addition, binding sites on the extracellular domain functionality of fAABs were identified, and IgG1 and/or IgG3 antibodies were found to be related to fAABs.

**Conclusion:**

Our data suggest that fAABs against GPCR play an essential role in vascular activity in chronic SM (hepatic and PAH) and might be involved in the development of hypertensive forms of SM.

## Introduction

Schistosomiasis (SM) is a parasitic disease caused by trematoda of the species *Schistosoma mansoni*. At least 800 million individuals in tropical regions worldwide are at risk of infection (1). This disease is the third most prevalent endemic parasitic disease associated with pulmonary hypertension in the world (2). Approximately 10% of infected individuals will present with the hepatosplenic clinical form, a fundamental condition for the development of pulmonary hypertension (PAH); among those presenting with the hepatosplenic form, 8–10% will develop PAH (3–5).

Parasite transmission is dependent on social factors, such as the presence of eggs of *S. mansoni*, the gastropod *Biomphalaria* in fresh water, and failure in populational educative strategies (6). Individuals infected by the parasite present with dermatitis, fever, and toxemic manifestations during the acute phase, as well as digestive and/or hepatointestinal, hepatosplenic, pulmonary arterial hypertension (PAH), glomerulonephritis, and neuroschistosomiasis during the chronic phase (7, 8). The main chronic manifestations of SM are related to the presence of *S. mansoni* eggs in distinct human organs, in which the immune response moves toward the egg, causing host tissue destruction and collagen/fibrosis deposition in a granulomatous process (9, 10).

PAH is a rare disease characterized by increased pressure in the pulmonary circulatory system, and consequent right atrial and ventricular overload. It is hemodynamically defined as mean pulmonary artery pressure ≥ 20 mmHg at rest with an occlusion pulmonary pressure < or = 15 mmHg (11).

Pulmonary reactions against *S. mansoni* eggs do not fit all the described vascular remodeling changes. Severe portal hypertension has been reported in patients with minimal liver fibrosis. However, when imaging techniques or histological evaluations are performed, they do not match the portal hypertension severity diagnosis (8, 12–14). A marked increase in hepatic and PAH resistance is a primary factor in portal hypertension and pulmonary development, respectively. Some of the liver and lung alterations are caused by vascular occlusion in the egg, embolization, and obstruction, which distort the liver architecture. This causes fibrosis, vascular remodelling, sinusoidal and vascular capillarization, and an unreversed increase in microcirculatory resistance. These dynamic mechanisms are modulated by adrenergic, renin-angiotensin and endothelin, and inflammatory systems, mediated by vasoactive neurotransmitters and peptides such as norepinephrine, angiotensin II, endothelin I, TGF-β, Th1-, Th2, Th17-like cells and cytokines. This causes vasodilatation/vasoconstriction misbalancing, leading to an increase in hepatic and pulmonary vascular tone (2, 15–18). Antibodies such as immunoglobulin (Ig)G1 and IgG4 are anti-*S. mansoni* antigens; thus, they have gained attention in the context of vaccine strategies (19, 20) and have been identified as key contributors to the worsening clinical prognosis of SM (21–23). However, in the presence of *S. mansoni*, self-reactive antibodies or fAABs can cause unexpected reactions in experimental and human tissues, resulting in adverse disturbances (24–26).

This study aimed to investigate and characterize the generation of fAABs/GPCRs in patients with SM presenting with acute and chronic (hepatic and pulmonary) forms of the disease.

## Materials and methods

### Patients

This was a cross-sectional comparative study. All subjects were admitted to the Reference and Treatment Center on Parasite and Infectious Diseases – “Orestes Diniz,” from the Faculty of Medicine of Federal University of Minas Gerais (UMFG), Belo Horizonte, MG, Brazil. Written informed consent was obtained from all patients, and the study was approved by UFMG Research Ethics Committee (CAAE-25095814.0.0000.5149 COEP-UFMG) and conducted according to the guidelines in the Declaration of Helsinki (2013) from the World Medical Association. *S. mansoni*-infected individuals were evaluated using clinical and laboratory tests (which tested for biochemical, neurohormonal, and inflammatory markers) and two-dimensional echocardiography with Doppler assessment of (i) pulmonary artery pressure levels and (ii) ventricular function.

Individuals were grouped as (i) hepatointestinal SM (Group 1, n = 10), hepatosplenic SM without pulmonary hypertension (Group 2, n = 14), hepatosplenic SM with pulmonary hypertension (Group 3, n = 7), acute SM (Group 4, n = 5), and uninfected individuals (Group 5, n = 10).

#### Inclusion criteria for SM-infected individuals

Individuals aged 18–60 years with a positive diagnosis for SM (determined via parasitological, histological, and/or serologic tests), presence or absence of periportal fibrosis and portal hypertension on ultrasound, absence of other liver diseases, and presence or absence of pulmonary hypertension on echocardiography (systolic pulmonary pressure > 35 mmHg).

#### Exclusion criteria for SM-infected individuals

Presence of diseases suggested by clinical history and complementary examinations including heart failure, rheumatic valve disease, acute myocardial infarction, congenital heart disease, history of pulmonary hypertension, pericardial disease, severe hypertension (stage 3), liver cirrhosis, renal failure, hypothyroidism, COPD, significant anemia, and pregnancy may be confounding factors in the interpretation of the etiology of pulmonary hypertension and pregnancy (elevated BHCG).

#### Criteria for the control group

Subjects aged 18–60 years, with no hospitalization in the last six months and negative serology for SM (uninfected individuals), were referenced as the control group of this study.

### Sample collection

Two mL of blood was obtained by venipuncture of the antecubital vein. The blood was immediately centrifuged, and the sera were isolated and stored at -80°C in an ultra-freezer for later evaluation of the activity of the anti-second loop of G Protein receptor, anti-endothelin 1 (ETA1), anti-angiotensin II receptor (AT1), and anti-α−1 adrenergic receptor.

### Immunoglobulin preparation

Immunoglobulin G (IgG) was originally isolated from patients’ sera, as described previously (27), before human IgG preparation. Briefly, sera (0.5 mL) were precipitated with 0.33 mL saturated ammonium sulfate, mixed, and incubated overnight at 4°C. Subsequently, the samples were centrifuged at 3,376*x*g and the supernatants were discarded and, the pellet was dissolved in 0.5 mL physiologic NaCl solution containing phosphate buffer (pH 7.4). Ammonium sulfate (0.5 mL) was added to precipitate the IgGs, and the sample was centrifuged. The resulting pellet was dissolved in 0.5 mL of the buffer (154mM Nacl, 10mM sodium phosphate, pH 7.2) and dialyzed in 1 L of the same buffer, at 4°C. The dialyze buffer was changed 4 times in 4 days. The crude antibody fraction (IgG) was stored at -20°C and later used in the cardiomyocyte bioassay. The estimated optimal pharmacological dilutions of the crude antibody fraction were 1:50, according to 28.

### Cardiomyocyte cell culture

Neonatal rat cardiomyocytes were isolated from the cardiac ventricles of 1–3-day-old Wistar rats (27). The procedures were approved by the Ethics Committee in Research (# Y9008/12 and # Tötungsanzeige Y9004/19) at the Max Delbrück Centre for Molecular Medicine Berlin, Germany.

Briefly, ventricles were dissected into 1-mm^2^ fragments with two scalpels in a Ca^2+^-free phosphate buffered saline (PBS) solution. After washing, the fragments were transferred to 10 mL of Ca^2+^-free PBS containing 0.2% crude trypsin. The heart fragments were slowly stirred using a magnetic stirrer for 15 min at 37°C. Then, the supernatant was added to a plastic tube containing 5 mL of ice-cold neonatal calf serum and centrifuged at 130*x*g for 15 min. Subsequently, the supernatant was discarded and the pellet was dissolved in complete SM 20-I cell culture medium. This procedure was repeated three times. The 4-cell containing SM 20-I samples were collected and centrifuged again. The new pellet was dissolved in a complete SM 20-I culture medium containing 0.5 µM fluorodeoxyuridine to prevent the over-growth of the non-myocytes. The isolated cells were seeded in culture flasks (12.5 cm^2^) at a density of 2.4 x 10^6^ cells/2 mL. The culture medium was changed after 24 h and then every second day. The cardiomyocytes started spontaneously beating after two days of culture and these cells were cultured for 4–10 days at 37°C.

### Cultured rat cardiomyocytes as “bioassays”

The beating rate of the spontaneously beating cardiomyocytes was measured on a heated desk (37°C) of an inverted microscope. First, the basal beating rate of cardiomyocytes was monitored at six marks on the culture flask. Then, immunoglobulins were added to the cell culture medium, and the beating rate was measured again at the corresponding mark after 5 or 60 min. The effects of the addition of specific receptor antagonists were also evaluated in this cell culture setting where α1adrenoceptors were blocked by 1 µM urapidil (Biomol, Germany), endothelin-1 receptor was blocked by 0.1 µM BQ123 (Tocris, Germany), and anti-angiotensin II AT1 receptors were blocked by 1 µM Losartan (Sigma, Germany). To identify the binding sites of fAABs on extracellular structures, fAABs were pretreated with peptides corresponding to the first, second, or third extracellular loops of the corresponding GPCR (data not shown). These experiments have shown that the fAABs recognize the second extracellular loop as binding site. Therefore, we used it for the estimation of the specific epitope of the fAABs on the second extracellular loop 5 to 6 short overlapping peptides representing this extracellular domain. The peptide that neutralizes the agonistic activity of fAABs represents the binding site or epitope of this loop.

### Statistical analysis

All data in this study were presented as mean ± standard error of the mean. Owing to our small sample size, we performed a pairwise non-parametric Kruskal–Wallis test or, a t-student test to examine statistical significance (*p<0.05*).

## Results

In this study, we identified and characterized the fAAB patterns in individuals with acute and chronic SM. In the sera of chronic subjects, three fAABs against GPCR were identified recognizing α1-adrenoceptor, endothelin-1, and angiotensin AT1 receptors.

**Figure 1:**
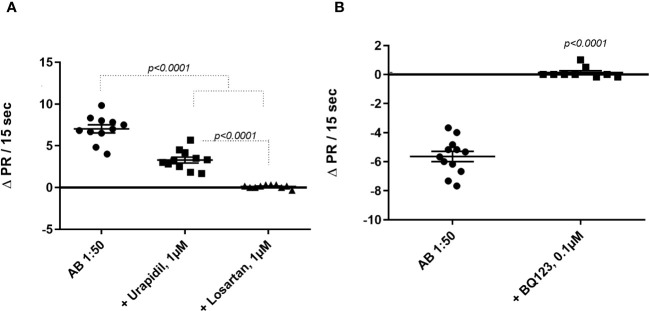
Inhibition of functional autoantibody – fAAB (1:50) through antagonists of **(A)** the α1-adrenoceptor (urapidil) 1µM and (ii) the angiotensin receptor (losartan) 1µM and **(B)** the endothelin receptor (BQ123) 0.1 µM. The graphic shows rat cardiomyocytes’ beating rate changes (delta pulse response/ΔPR/min) in the presence of all antagonists. Statistical differences were informed in the graphic when *p<0.05*.

**Figure 2:**
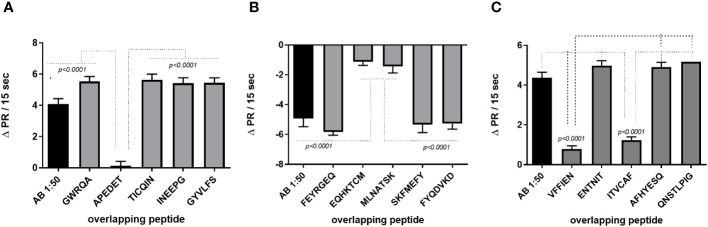
Activity of functional autoantibody in the sera of infected individuals diagnosed with Schistosomiasis (SM). Functional antibodies act against α1-adrenergic **(A)**, endothelin-1 **(B)** and angiotensin-II **(C)** AT1 receptors. The graphics represent the cardiomyocytes beating rate changes (delta pulse response/ΔPR/min) after stimulation with sera from uninfected individuals, from individuals in the acute phase of SM and, in the chronic phase of SM with hepatointestinal and hepatosplenic forms with and without pulmonary hypertension (PHT). Statistical differences among clinical forms were expressed in the graphic by *p<0.05*.

The agonist-like effects triggered by fAABs were blocked by specific antagonists of the α1-adrenoceptor (1 µM urapidil) and angiotensin-II AT1 receptors (1 µM losartan) (**Figure 1A**) and, of the endothelin-1 receptor (0.1 µM BQ123) (**Figure 1B**). In addition, we identified fAABs in the sera of SM individuals with PAH and hepatointestinal clinical forms. In those individuals in the acute phase of SM, only fAABs against α1-adrenergic (**Figure 2A**) and endothelin-1 (**Figure 2B**) receptors were changed, but not fAABs against angiotensin-II AT1 receptor (**Figure 2C**). There were no fAABs against GPCR in the uninfected subjects.

**Figure 3:**
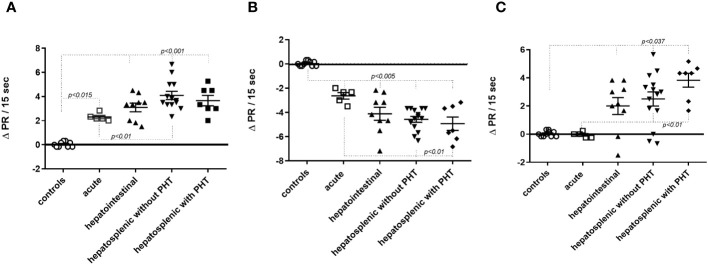
Identification of binding sites on the distinct extracellular domains of functional autoantibody (diluted 1:50) against the α1-adrenoceptor **(A)**, the endothelin-1 receptors **(B)**, and the angiotensin II (AT1) receptors **(C)**. Statistical differences were informed in the graphic when *p<0.05*.

In another set of experiments, we evaluated the binding sites of fAABs on the second extracellular loop of the receptors and IgG subclasses. **Figure 3A** shows the epitope analysis for the α1-adrenoceptor, whose fAABs were neutralized by the peptide APEDE, located in the second extracellular loop peptide of the adrenoceptor. The other short overlapping peptides did not exhibit significant inhibitory effects (**Figure 3A**). The endothelin-1 receptor was neutralized by peptides EQHKTCM and MLNATSK; however, since both overlapped, we assumed that against endothelin-1 receptors, fAABs recognized the largest epitope (**Figure 3B**). In contrast, fAABs anti-angiotensin II AT1 receptors were neutralized by the N-terminal part of the second extracellular loop of the peptide VFFIEN and, by one peptide of the middle region of the second extracellular loop ITVCAF (**Figure 3C**). The other overlapping short peptide in the second extracellular domain of the AT1 receptor did not have a significant effect.

**Figure 4:**
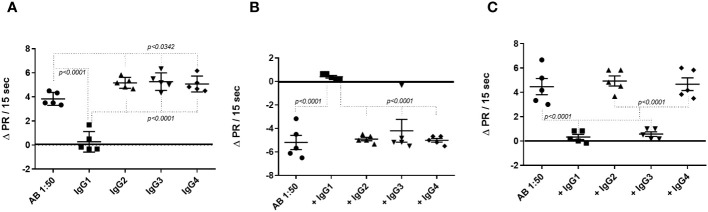
Determination of the IgG subclasses functional autoantibody (fAAB) diluted 1:50 against the GPCR. Cells were pretreated with antagonists of α1-adrenoceptor (urapidil) 1µM **(A)** and of endothelin receptor, BQ123 0.1 µM **(B)** and, fAABs were neutralized by anti-human IgG1 subclass. However, when cells were pretreated with the antagonist of angiotensin receptor (losartan) 1µM **(C)**, the fAABs were neutralized by anti-human IgG1 and IgG3 **(C)**. Statistical differences were informed in the graphic when *p<0.05*.

Next, the three identified fAABs were precipitated with monoclonal mouse anti-human antibodies belonging to the IgG1, IgG2, IgG3, and IgG4 subclasses. An anti-mouse polyclonal antibody was added to enlarge the mouse anti-human antibody complex. This antibody complex was centrifuged at 16,030*x*g and the supernatant was used in the experiments. As shown in **Figure 4A**, the activity of the α1-adrenoceptor fAABs was neutralized by mouse anti-human antibodies of the IgG1 subtype. In contrast, AT1 receptor fAABs were neutralized by mouse anti-human IgG1 and IgG3 antibodies (**Figure 4B**). The activity of the agonist-like AAB against the ETA1 receptor was blocked by anti-human IgG1, except in one patient who developed the IgG3 subtype fAAB (**Figure 4C**).

## Conclusion

In the current investigation, individuals chronically infected by *S. mansoni* developed fAABs against three different GPCRs and, in this sense, we evaluated them in the presence of agonist-like fAABs against α1-adrenoceptor, ETA1, and AT1 receptors.

GPCRs are seven-transmembrane receptor domains that can use GTP-binding proteins for signal transduction and are involved in various physiological and pathophysiological processes. Studies have demonstrated the participation of GPCRs in tropical disease agents such as protozoan and trematode infections (29, 30). However, no investigation has been performed to identify fAABs targeting GPCRs in (acute and chronic) SM, or whether they exert disturbances in distinct clinical forms of the disease.

Individuals in acute SM were able to generate fAABs against the α1-adrenoceptor and ETA1 receptor, but not against the AT1 receptor. The activity of fAABs was significantly lower than that of chronic SM. Interestingly, the same fAAB pattern was observed in individuals diagnosed with pulmonary hypertension without SM (31). It seems that vasoactive fAABs, like α1-adrenoceptor or endothelin ETA-receptor, may play an important role in pulmonary hypertension pathogenesis, since the removal of such fAAB by immunoadsorption promotes a fast reduction in the dilated diameter of the right atrium and ventricle, reducing the resistance of the pulmonary vessels and increasing the oxygen saturation. Symptoms of PAH have also been observed in individuals chronically infected with *S. mansoni*. Therefore, we assume that the vasoactive fAABs against the α1-adrenoceptor and the endothelin-1 ETA receptor were important players in PAH related to SM, since fAABs activate the receptors as classical agonists. Moreover, these fAABs could be used as an early marker for patients with pulmonary arterial hypertension, in the late phase of SM. In addition, fAABs (eg. GPCR-autoantibodies) can permanently stimulate cells without desensitization or internalization of receptors (32).

Additionally, a third vasoactive fAAB that recognized the angiotensin II (AT1) receptor was associated with chronic hepatosplenic individuals. This idea is supported by the observation of AT1 receptor activation in inflamed and ischemic kidney arteries in rats (33). In healthy kidney arteries, the authors demonstrated that fAABs against the AT1 receptor did not have any effect, although angiotensin II was able to activate this receptor. AT1 fAABs may also play an important role in preeclampsia and kidney diseases (34).

Moreover, AT1- and α1-adrenoceptor fAABs can maturate and degranulate mast cells in cultured neonatal rat heart cardiomyocytes which can release several cytokine and enzymes such as TNF, chymase, tryptase, and histamine (35). Additionally, AT1 receptor fAABs increase the adhesion of human monocytes to endothelial cells and induce the formation of tissue factors in other immune cells. Both effects were blocked by losartan, an AT1 receptor antagonist, and were observed in monocytes of normotensive and hypertensive individuals (36). Previous studies showed that fAABs against the AT1 receptor elevated the expression of tissue factor (TF) in monocytes, an essential element of the coagulation cascade. Furthermore, AT1 receptor fAABs have also been identified in preeclamptic patients, causing activation and expression of TFs in vascular cells (37) and in thrombocytes, mediating signalling to platelet-leukocyte-endothelial cell interactions (38).

The fAABs may signalize the presence and the intensity of the immune response and, consequently, can act as biomarkers of prognosis in autoimmune diseases (type 1 diabetes, celiac diseases, thyroiditis) or even in cardiovascular and parasite evolutive diseases (39–41). The fAABs can be identified in biological samples years before the development of the disease, which means they should be able to predict diseases. The goal of predicting diseases is the possibility of preventive intervention using pharmacology or other biotechnologies to block their actions. The matter of fact is that understanding the role and specificity of fAABs in distinct non-autoimmune diseases, such as what we are showing in schistosomiasis, opens a new window of clinical opportunity. However, for a conclusive clinical application of fAABs, three parameters must be quantified – identification of its sensitivity and specificity of prediction and, of its positive predictive values.

Altogether, beyond *Schistosoma* egg embolization with portal and pulmonary circulation obstruction, there will be focal arteritis, vessel destruction, and plexiform lesions. These pathological conditions suggest the release of several cytokines and neurohormones from the adrenergic, renin-angiotensin, and endothelin systems. In addition, vascular heparan sulfate proteoglycans and endothelial adhesion molecules may be overexpressed after fAAB activity, contributing to an increase in (i) vascular resistance, (ii) vasoconstriction, (iii) coagulation processes with microclot formation, and portal and PAH (42). Therefore, this study assumed that individuals with chronic schistosomiasis produce fAABs against GPCR, whose activity might be involved in the development of hypertensive forms of the disease. Under *S. mansoni* infection conditions, fAAB can disturb the neurohormonal balance and represents a key player in the pathophysiological development of the liver and pulmonary clinical forms of SM.

## Data availability statement

The raw data supporting the conclusions of this article will be made available by the authors, without undue reservation.

## Ethics statement

The studies involving humans were approved by UFMG Research Ethics Committee (CAAE-25095814.0.0000.5149 COEP-UFMG) and conducted according to the guidelines in the Declaration of Helsinki (2013) from the World Medical Association. The studies were conducted in accordance with the local legislation and institutional requirements. The participants provided their written informed consent to participate in this study. The animal study was approved by Ethics Committee in Research (# Y9008/12 and # Tötungsanzeige Y9004/19) at the Max Delbrück Centre for Molecular Medicine Berlin, Germany. The study was conducted in accordance with the local legislation and institutional requirements.

## Author contributions

FB: Conceptualization, Methodology, Resources, Visualization, Writing – original draft, Writing – review & editing. JL: Conceptualization, Formal analysis, Methodology, Writing – original draft, Writing – review & editing. RS: Data curation, Formal analysis, Funding acquisition, Methodology, Writing – original draft, Writing – review & editing. JM: Conceptualization, Formal analysis, Funding acquisition, Methodology, Project administration, Writing – original draft, Writing – review & editing. AT: Formal analysis, Funding acquisition, Methodology, Visualization, Writing – original draft, Writing – review & editing. GW: Conceptualization, Formal analysis, Funding acquisition, Methodology, Resources, Writing – original draft, Writing – review & editing.
